# Supplementary material for: Shared Treg dysregulation underlies psoriasis vulgaris and atopic dermatitis through the PHF19-PRC2 epigenetic axis
Source: Front Immunol. 2026 Aug 20;17:1932788. doi: 10.3389/fimmu.2026.1932788 (PMC13538097; doi:10.3389/fimmu.2026.1932788)
Supplement: Supplementary file 3 [file DataSheet3.zip › Additional files/Additional file 6/48.CTSS.forest.pdf]

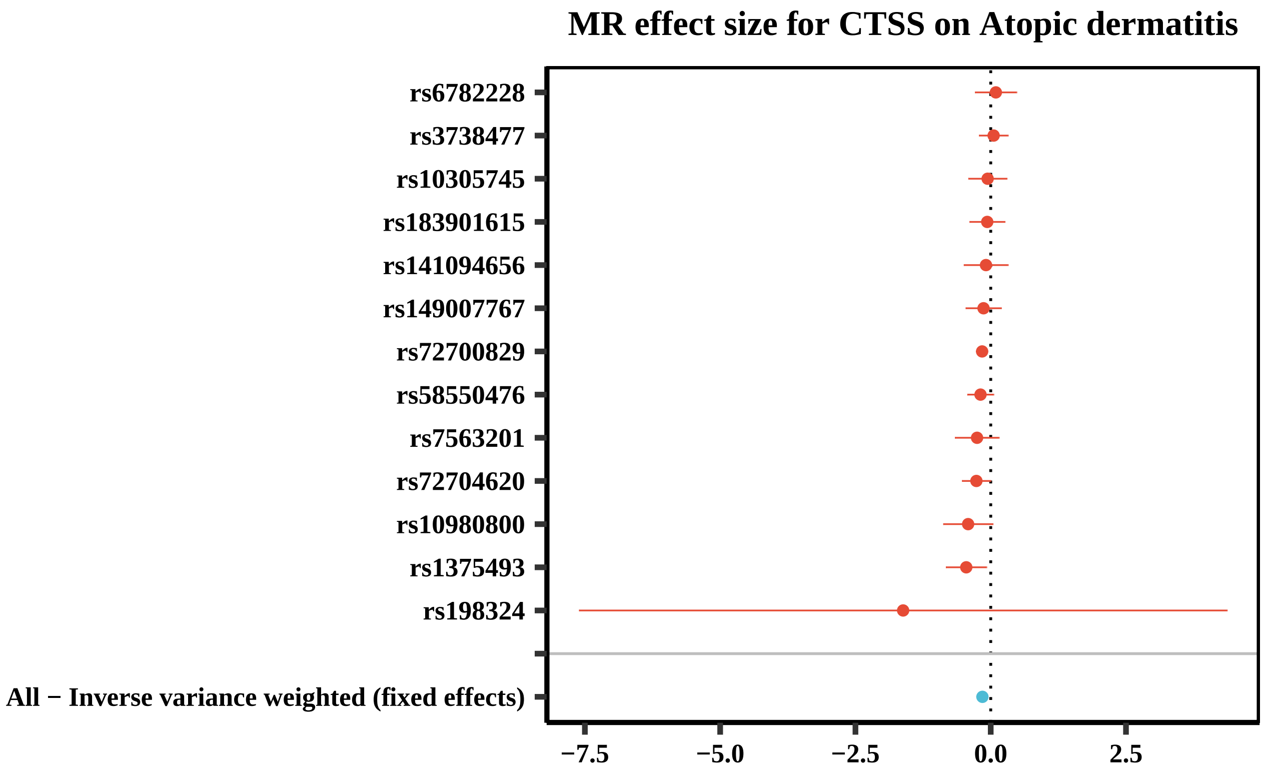
MR effect size for CTSS on Atopic dermatitis
rs6782228
rs3738477
rs10305745
rs183901615
rs141094656
rs149007767
rs72700829
rs58550476
rs7563201
rs72704620
rs10980800
rs1375493
rs198324
All − Inverse variance weighted (fixed effects)
−7.5
−5.0
−2.5
0.0
2.5
